# Conditional Knockout of Integrin α2β1 in Murine Megakaryocytes Leads to Reduced Mean Platelet Volume

**DOI:** 10.1371/journal.pone.0055094

**Published:** 2013-01-24

**Authors:** David Habart, Yann Cheli, Diane J. Nugent, Zaverio M. Ruggeri, Thomas J. Kunicki

**Affiliations:** 1 Roon Research Center for Arteriosclerosis and Thrombosis, The Department of Molecular and Experimental Medicine, The Scripps Research Institute, La Jolla, California, United States of America; 2 Hematology Research, CHOC Children's Hospital, Orange, California, United States of America; Royal College of Surgeons, Ireland

## Abstract

We have engineered a transgenic mouse on a C57BL/6 background that bears a floxed *Itga2* gene. The crossing of this mouse strain to transgenic mice expressing Cre recombinase driven by the megakaryocyte (MK)-specific Pf4 promoter permits the conditional knockout of *Itga2* in the MK/platelet lineage. Mice lacking MK α2β1 develop normally, are fertile, and like their systemic α2β1 knockout counterparts, exhibit defective adhesion to and aggregation induced by soluble type I collagen and a delayed onset to low dose fibrillar collagen-induced aggregation, results consistent with blockade or loss of platelet α2β1. At the same time, we observed a significant reduction in mean platelet volume, which is consistent with the reported role of α2β1 in MK maturation and proplatelet formation in vivo. This transgenic mouse strain bearing a floxed *Itga2* gene will prove valuable to distinguish *in vivo* the temporal and spatial contributions of α2 integrin in selected cell types.

## Introduction

The integrin α2β1 (VLA-2; GPIa-IIa, CD49b) is widely expressed on numerous cell types and binds specifically to type I collagen and decorin [1], [2], but also to collagens types II-V and laminins 1 and 5 [3]. The α2 protein was first isolated from platelets where it mediates adhesion to extracellular matrix collagen and contributes to the initiation of platelet activation and hemostasis [4], [5]. α2β1 also plays an important role in megakaryocyte (MK) maturation, where it mediates MK binding to collagen I in the bone marrow thereby delaying proplatelet formation through mechanisms dependent on Rho-ROCK and other pathways [6], [7], [8].

The analyses of α2β1 deficiency in two mouse models to date have utilized systemic *Itga2*−/− mice [9], [10]. In both models, in vitro platelet responses initiated by soluble collagen were impaired, but no obvious in vivo hemostatic defects were observed. In one study, normal platelet counts were also reported for systemic *Itga2*−/− mice [10].

One problem of systemic knockout mouse models is that compensating effects within and between different cell lineages can obscure tissue-specific effects. This may be particularly true in the case of a ubiquitous receptor such as α2β1, which is expressed in the various cellular compartments of the circulation (platelets, mononuclear cells), the vasculature (endothelial cells, mural cells, fibroblasts) and the bone marrow (megakaryocytes, myeloid precursors, stromal cells). The conditional knockout of such ubiquitous proteins, using methods such as the Cre-Lox system, can help to distinguish the variety of effects contributed by these various cell types.

For these reasons, we engineered a transgenic mouse on a C57BL/6 background that bears a floxed *Itga2* gene. The crossing of this mouse strain to transgenic mice expressing Cre recombinase driven by the MK-specific Pf4 promoter permits the conditional knockout of *Itga2* in the MK/platelet lineage.

In this report, we evaluate selected functions and relevant physical characteristics of platelets from conditional MK *Itga2*−/− mice.

## Materials and Methods

### Ethics Statement

This study was conducted in strict accordance with the recommendations in the Guide for the Care and Use of Laboratory Animals of the National Institutes of Health. The protocol was approved by the Institutional Animal Care and Use Committee of The Scripps Research Institute (Assurance of Compliance No. A3194-01) and CHOC Children's Hospital (Assurance of Compliance No. A3987-01).

### Engineering of the loxP-Itga2 mouse

**Figure 1:**
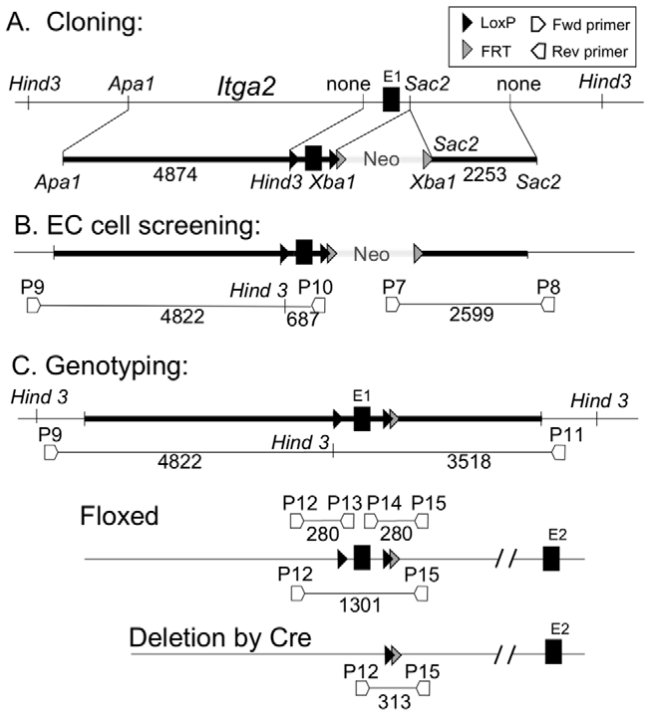
Engineering of the loxP-*Itga2* transgene. **A**) The targeting vector construction is depicted schematically to show positions of the key restriction sites used to clone exon1 flanked by loxP sites (middle), long arm (left) and short arm (right) DNA segments into the vector pBS-FRT-neo-FRT, which already contained the Neomycin-Kanamycin resistance marker flanked by FRT sequences, removable by Flp-recombinase. The positions of loxP and FRT sites are shown relative to exon 1 (E1). A unique Hind 3 restriction site was introduced next to the loxP sequence to facilitate the verification of a correct homologous recombination event in later stages. **B**) The screening strategy to identify ES cells with a correct recombination event. Two PCR reactions were employed, each utilizing one primer located within the natural α2 gene sequence beyond the end of the short (P8) or long (P9) arms, thus excluding amplification of random insertions. **C**) Genotyping strategy employed to detect removal of the Neomycin cassette by Flp-recombinase using primer pairs P12/P13 and P14/P15. Once homozygous mice were obtained, the correct location of the trans-gene was verified by PCR amplification of the entire region using primer pair P9/P11, each located in the natural *Itga2* gene sequence beyond the long and short arms, followed by *Hind3* restriction fragment length polymorphism analysis. Cre-recombinase mediated removal of the floxed exon 1 was confirmed by PCR using primer pair P12/P15.

A targeting vector was designed to add loxP sites flanking exon 1, which contains 149 bp of the 3′ UTR and the first 19 codons (**Figure 1**). The deletion of exon 1 had previously been shown to be sufficient to inactivate *Itga2* [9]. The long and short arms were designed to avoid repetitive sequences using the online application RepeatMasker (http://www.repeatmasker.org/cgi-bin/WEBRepeatMasker). Naturally occurring *Apa1* and *Sac2* sites in the long and short arms were used for cloning. The short arm and exon 1 were amplified from Bruce 4 ES cell gDNA, and the long arm was amplified from BAC PR23-448L13 (Children's Hospital Oakland Research Institute), using Phusion High-Fidelity DNA polymerase (Thermo Scientific, Inc., Vantaa, Finland). The floxed exon 1, the short arm and the long arm respectively were cloned into pBS-FRT-Neo-FRT (a gift from Dr. Uli Mueller, TSRI) in three stages. First, the floxed exon 1 (954 bp) was cloned directionally between *Hind3* and *Sma1* sites of the vector, thus introducing a novel *Hind3* site to serve as a genotyping marker. Second, the short arm (2253 bp) was cloned in the *Sac2* site. Finally, the long arm (4783 bp) was cloned directionally between *Apa1* and *Hind3* sites. The correct sequence was confirmed by Sanger sequencing. The plasmid was harvested using the EndoFree Maxiprep kit (Qiagen, Valencia, CA). Plasmid DNA (180 ug) was linearized with *Apa1*, precipitated with ethanol and submitted to the Mouse Genetic Core at TSRI for Bruce 4 ES cell electroporation. Stably transfected cells were selected by *Neo* resistance. A total of 304 Bruce 4 ES cell clones were screened for homologous insertion of the transgene by PCR across the short and the long arms using primer pairs P7/P8 and P9/P10 (*Table 1*) and *Hind3* restriction fragment length analyses.

**Table 1:** PCR Primers.

Name	Direction	Sequence
P7	Forward	5′-TCGCCTTCTTGACGAGTTCT-3′
P8	Reverse	5′-GGAAGGCCAGGTTCAAAGTT-3′
P9	Forward	5′-TTTCAAAAACTCAGATATAAGACTCCA-3′
P10	Reverse	5′-TCCCTCCCTGGTCTTCTAGG-3′
P11	Reverse	5′-GGAGAGAATGAGGGATTGACCT-3′
P12	Forward	5′-GTGTCGAACCTGGTCATTCC-3′
P13	Reverse	5′-TCTAGCTCACCAGACCCAAGA-3′
P14	Forward	5′-TCACACCCACAGATTTGGAG-3
P15	Reverse	5′-CGGTTCCTGGTTCAGAGCTA-3′
Pf4-CreF	Forward	5′-CCCATACAGCACACCTTTTG-3′
Pf4-CreR	Reverse	5′-TGCACAGTCAGCAGGTT-3′

**Figure 2:**
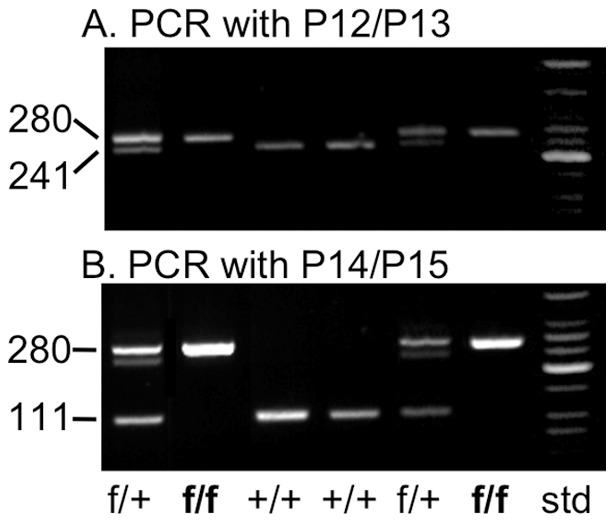
PCR to distinguish floxed (f) and wild type (+) *Itga2*. **A).** Using primer pair P12/P13 (sequences listed in Table 1), the size of the wild-type PCR product is 241-bp; that of the floxed PCR product is 280-bp. **B**) Using primer pair P14/P15, the wild-type product is 111-bp; that of the floxed *Itga2*, 280-bp. std  =  DNA size standards.

Two Bruce 4 ES cell clones (#103 and #268) were injected into (BALB/c ByJ x B6(Cg)-Tyr<c-2J>/J)F2 blastocysts, performed by the TSRI Mouse Genetics Core. From clone #103, ten chimeric mice were born, and two males with a black coat contribution greater than 50% were selected for further breeding. From clone #268, twenty-nine chimeric mice were born, and nine with a black coat contribution greater than 50% were selected for further breeding. In 9 of the 11 chimeric males, the presence of the correctly inserted long arm was confirmed by PCR. All eleven males were crossed to female albino B6 mice imported from The Jackson Laboratory (JAX; Bar Harbor, Maine) designated B6(Cg)-Tyr<c-2J>/J (JAX #000058) to determine germ line transmission by coat color in the resulting pups. The presence of the floxed *Itga2* gene in black pups was confirmed by PCR, and mice with the correct recombination were crossed to deletor (Flp) albino B6 mice (B6;SJL-Tg(ACTFLPe)9205Dym/J; JAX #003800; N13-N14 against albino B6 mice), so that the selection cassette flanked by the FRT sequences was removed, as confirmed by PCR, using primer pair P9/P11 (**Table 1**). The resulting mice were intercrossed to obtain α2^flox/flox^ homozygotes, and genotype was confirmed by PCR with primer pairs P12/P13 and P14/P15 (**Figure 2**). Our floxed-*Itga2* strain has been deposited in The Jackson Laboratory collection and is designated C57BL/6-*Itga2*<tm1Tkun>/J (JAX #018921).

### Conditional α2 knockout mice (α2-cKO)

Transgenic mice expressing the codon-improved Cre recombinase under the control of the mouse platelet factor 4 (Cxcl4) promoter (Pf4-Cre) [11] (C57BL/6-Tg(Pf4-cre)Q3Rsko/J; JAZ #008535) were crossed with our α2^flox/flox^ mice to generate heterozygous α2^flox/+^; Pf4-Cre+ mice. α2-cKO mice were produced from crosses of α2^flox/−^; Pf4-Cre+ mice x α2^flox/flox^ mice. Genotyping was performed in house, using primers listed in **Table 1**: The primer pair Pf4-CreF/Pf4-CreR yields a 450-bp product; The primer pair P12/P15 yields a 313-bp product from the Cre-excised α2^flox^ gene and a 1301-bp product from the non-excised α2^flox^ gene.

### Platelet parameters

Peripheral blood platelet counts and mean platelet volume (MPV) were analyzed as previously described [12], using a Cell-Dyn Emerald apparatus (Abbott Laboratories, Abbott Park, IL). Expression of platelet receptors, including GPIbα, GPVI, and integrins α2β1, α5β1 and αIIbβ3 was measured by flow cytometry as described [13], using a FACSCalibur flow cytometer (Becton, Dickinson and Company, Franklin Lakes, NJ).

### Western Blots

Tissue samples (lung, spleen) from α2 cKO mice and α2^flox/flox^ littermates were minced, homogenized in TRIS lysis buffer containing 2% SDS and protease inhibitors. Particulate and non-soluble material were removed by centrifugation, and soluble proteins from each source were separated by SDS-PAGE under reducing conditions and transferred to nitrocellulose membranes [14]. Platelet and mononuclear cells (MNC) were isolated from heparanized peripheral blood, as described [13], [15], and MNC were further cleared of platelets by absorption with polyclonal anti-mouse CD41 coupled to magnetic beads. Purified platelets and MNC were each solubilized in SDS buffer, proteins were separated by SDS-PAGE and transferred to nitrocellulose membranes. Membranes were blocked and incubated with polyclonal rabbit anti-mouse α2 antibody. Bound antibody was detected by HRP-conjugated anti-rabbit IgG and ECL (Amersham Biosciences) [14].

### In Vitro Platelet Function Tests

The aggregation of platelets in platelet-rich-plasma (PRP) and the adhesion of platelets to soluble type I human collagen (purified in our laboratory) or bovine tendon collagen (fibrillar; Sigma-Aldrich, St. Louis, MO) were performed as described [13], [16].

### Tail bleeding times

In vivo haemostatic function was assessed by tail bleeding times, as described [17].

### Statistical Analysis

Normally distributed variables were described by mean and standard deviation (SD), and the statistical differences were calculated by the t-test. A p-value ≤0.05 was considered statistically significant.

## Results

When α2^flox/flox^ mice were crossed to Pf4-Cre positive mice (Jackson Laboratories), the floxed exon 1 was removed. Primary mouse megakaryocytes (≥95% purity) as a source of gDNA were isolated from femur and tibia bone marrow as described by Senis et al. [18], and the excision of exon 1 in megakaryocyte gDNA from PF4-Cre+;α2^flox/flox^ mice was confirmed by PCR, using primer pair P12/P15 (**Figure** **1**).

### Phenotypic characterization of α2 cKO mice

**Figure 3:**
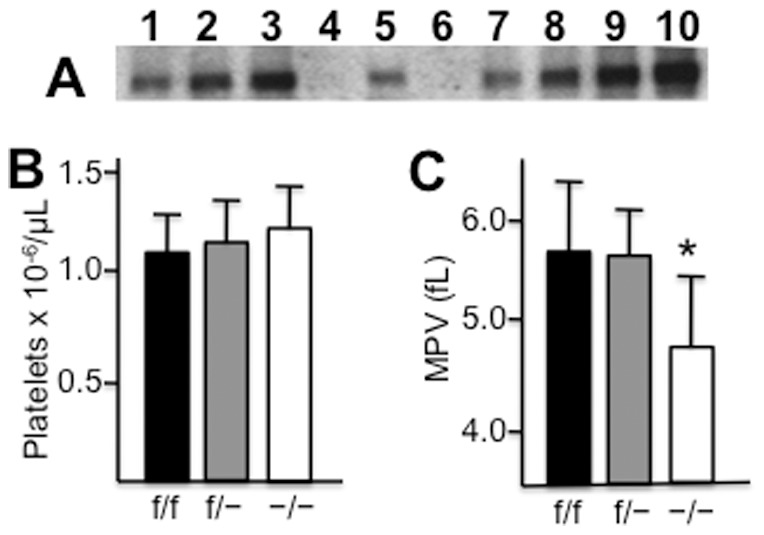
Comparison of Pf4-Cre(neg);α2^flox/flox^ mice (f/f), heterozygous Pf4-Cre(neg);α2^+/flox^ mice (f/−) and α2-cKO mice (−/−). (**A**) Detection by western blot of α2 integrin in equivalent total protein samples from mononuclear cells (1, 2), platelets (3 – 6), lung (7, 8) and spleen (9, 10). Purified proteins were analyzed from f/f mice (1,3,7,9), f/− mice (5) and −/− mice (2,4,6,8,10). (**B,C**) Platelet parameters. Platelet count (**B**) and MPV (**C**) were measured in peripheral whole blood from f/f, f/− and −/− mice. The mean +/− SD are depicted (n = 6 in each case). The difference in MPV between f/f and −/− mice was statistically significant (p<0.01) (*).

The complete loss of integrin subunit α2 in platelets of α2 cKO mice was confirmed by western blot of proteins extracted from platelets (**Figure 3A**). Using the same approach, we established that the content of integrin α2 in other tissues, including spleen, lung and blood mononuclear cells, of α2 cKO mice was normal (**Figure 3A**). These results confirm the conditional megakaryocyte/platelet knockout of the α2 subunit. As shown in **Figure 3B**, platelet counts in α2 cKO mice (designated as −/−) (123±19×10^−6^/μl; mean ± SD; n = 6) were somewhat higher than those of Cre-PF4(neg);α2^flox/flox^ mice (designated +/+) (111±18; n = 6), but the difference was not statistically significant (p = 0.264). However, MPV (**Figure** **3C**) for α2 cKO mice (4.7±0.7 fL; n = 6) was significantly lower (19% reduction) than that of Cre-PF4(neg);α2^flox/flox^ mice (5.8±0.7 fL; n = 6) (p = 0.029).

**Figure 4:**
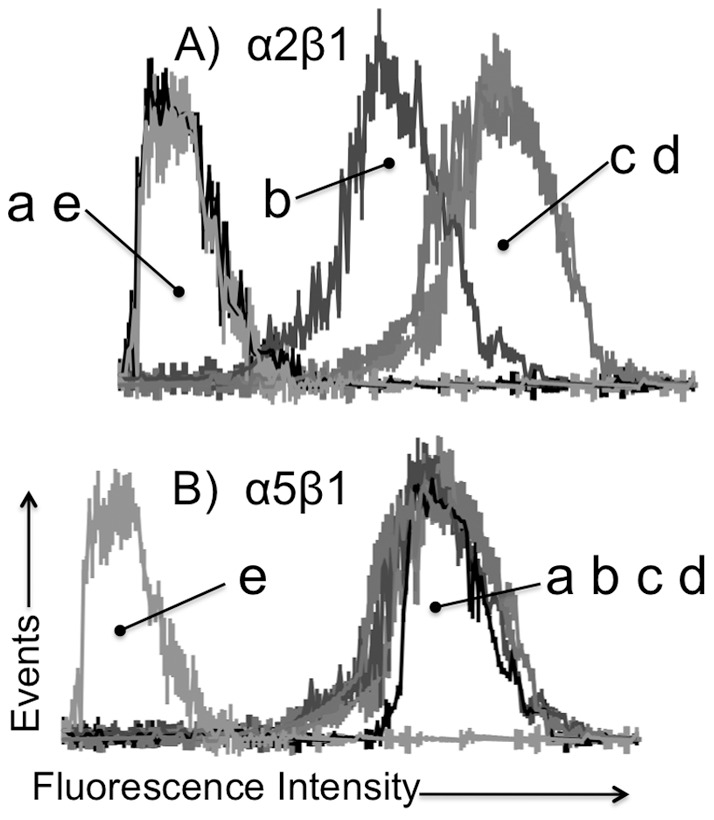
Platelet expression of integrins (A) α2β1 and (B) α5β1 determined by flow cytometry, using rat anti-mouse monoclonal antibodies. The results are representative of four separate experiments. Platelets from the following mice were compared: (**a**) Pf4-Cre+;α2^flox/flox^; (**b**) Pf4-Cre+;α2^flox/+^; (**c**) Pf4-Cre(neg);α2^flox/flox^; and (**d**) C57BL/6 mice. As a negative control, (**e**) depicts the binding of an isotype identical, non-reactive IgG1 monoclonal antibody to platelets from Pf4-Cre+;α2^flox/flox^ mice.

To evaluate platelet surface expression of α2β1, with reference to a second integrin α5β1, platelets in whole blood were analyzed by flow cytometry, as described [15], using anti-mouse integrin α2 (Sam.G4) and anti-mouse integrin α5 (Tap.A12) rat monoclonal antibodies (Emfrets, Germany) (**Figure 4**). Anti-α5 is an appropriate control because the levels of α2β1 and α5β1 on platelets from the inbred C57BL/6 strain are comparable [15]. Platelets from PF4-Cre+;α2^flox/flox^ mice express very low to undetectable levels of platelet α2β1 but normal levels of platelet α5β1. On the other hand, platelets from PF4-Cre(neg); α2^flox/flox^ mice express levels of α2β1 or α5β1 equivalent to normal inbred C57BL/6 mice. At the same time, platelets from heterozygous PF4-Cre+; α2^flox/+^ mice express roughly one-half the normal level of α2β1 and normal levels of α5β1. An istotype identical, irrelevant nonoclonal IgG failed to bind above baseline with any mouse platelet source. Additional analyses of Pf4- Cre+;α2^flox/flox^ mice platelets confirmed the exclusive loss of α2β1, but normal expression of other receptors, namely αIIbβ3, GPIbα and GPVI, using rat monoclonal antibodies Leo.D2, Xia.G5 and Gon.C2, respectively (Emfrets) (not shown). These results confirm that the introduction of the loxP sites flanking exon 1 of *Itga2* does not affect expression of integrin α2β1 on platelets and that the introduction of the PF4-Cre gene in vivo results in the unique excision of *Itga2* exon 1 in megakaryocytes and a specific loss of expression of platelet α2β1.

### Defective adhesion of α2 cKO platelets to soluble type I human collagen

**Figure 5:**
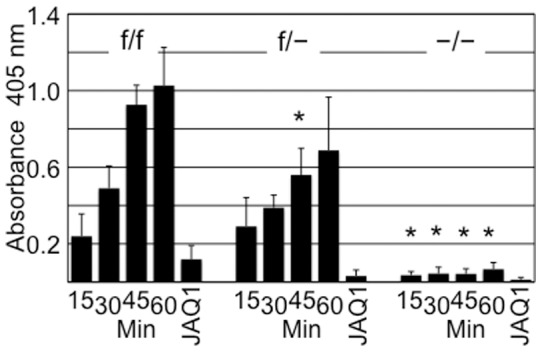
Platelet function: in vitro adhesion to soluble type I human collagen. Washed platelets from f/f, f/− and −/− mice were allowed to adhere under static conditions to soluble type I collagen immobilized on microtiter plates. Adherent platelets were quantitated by measuring Absorbance at 405 nm (ordinate) in the absence of inhibitory antibody at 15, 30, 45 and 60 minutes (abscissa) or in the presence of the GPVI inhibitory antibody JAQ1 at 60 minutes. The results of a single experiment, representative of three identical experiments, are depicted as the mean ± SD. The asterisks (*) denote results that are significantly different (p<0.5) from those obtained for f/f mice.

Platelets from f/f littermates adhere to type I human collagen, as expected, in the absence of the GPVI inhibitor antibody JAQ-1 (**Figure 5**). The inhibition by JAQ-1 confirms the importance of GPVI in platelet adhesion to collagen type I under static conditions. At the same time, even in the absence of JAQ-1, platelets from heterozygous f/- littermates exhibited a reduction in adhesion that was statistically significant at 45 minutes (p<0.05) and completely abolished in the presence of JAQ-1, while platelets from −/− mice exhibited negligible adhesion even in the absence of JAQ-1. These results confirm the importance of α2β1 in platelet adhesion to soluble collagen and demonstrate that the in vitro platelet function of our −/− mice is equivalent to that observed for systemic α2-deficient mice [10].

### Defective aggregation of α2 cKO mice by soluble collagen

**Figure 6:**
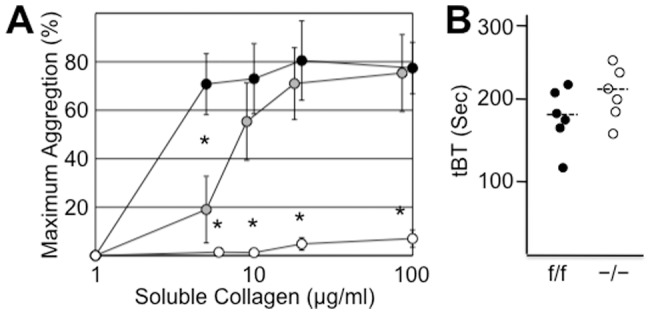
Platelet function. (**A**) In vitro platelet aggregation in response to soluble type I human collagen. Heparinized platelet-rich-plasma from f/f (black), f/− (gray) or −/− (white) mice was incubated with the indicated concentrations of soluble type I collagen. Results are expressed as the mean ± SD (n≥4). The asterisks (*) denote results that are significantly different (p<0.5) from those obtained for f/f mice. (**B**) In vivo tail bleeding time. Tail bleeding times (sec) are depicted for 6 individual mice in each group together with the mean for each group (− − −).

The aggregation of platelets induced by soluble collagen has been previously shown to be abnormal in systemic α2- or β1-deficient mice [10], [19]. Consistent with those findings, we observed that the aggregation of platelets from our −/− mice was abnormal relative to f/f littermates (**Figure** **6A**). Platelets from −/− mice showed a negligible response to soluble type I human collagen up to a concentration of 100 μg/ml. In contrast, for f/f littermates, half-maximal aggregation was obtained at a concentration <5 μg/ml; for heterozygous f/− littermates, ∼7 μg/ml. Platelets from our −/− mice exhibited normal maximal aggregation in response to fibrillar collagen (not shown), as previously reported for systemic KO mice [9], [10], although the lag time between addition of agonist and initiation of aggregation with low dose fibrillar collagen (1 μg/ml) was consistently delayed: for f/f mice, 27.3±5.8 sec (n = 4); for −/− mice, 41.5±7.7 sec (n = 4) (p<0.05). At a higher fibrillar collagen dose (10 μg/ml), the lag times were similar: 21.8±3.9 sec vs. 22.3±3.4 sec. Comparable findings have been obtained in the presence of α2β1-blocking antibodies [20] or in either systemic α2β1 KO [10] or β1 KO mice [19].

### Normal tail bleeding times

The effect of the conditional α2 knockout on the in vivo tail bleeding time (tBT) was also assessed. Although the difference was not statistically significant (p = 0.126), the tBT in −/− mice (208±30 sec; n = 6) was slightly higher than that of f/f mice (178±34 sec; n = 6) (**Figure 6B**).

## Discussion

Platelet count and mean platelet volume (MPV) are two fundamental properties of normal platelets that can have a significant impact on platelet function in vivo, and MPV is in an important risk factor for negative outcomes in coronary artery and cerebrovascular disease.

The genetic component of platelet size has been studied in humans taking advantage of inherited platelet disorders as well as genome-wide association studies, as recently reviewed [21]. An inverse association of the of *ITGA2* rs28095 minor allele T with mean platelet volume in patients with acute coronary syndrome was observed, suggesting that α2 integrin plays a direct role in the regulation of mean platelet volume [12].

In the present study we demonstrate that the α2 integrin influences the platelet size, using a novel genetically engineered mouse model. After removal of the alpha2 integrin selectively from the megakaryocyte lineage a decreased platelet size was observed. The mechanistic explanation for the role of α2 integrin platelet size is likely to involve its contribution to megakaryocyte maturation within the bone marrow.

According to a widely-held theory of platelet biogenesis in the bone marrow, immature megakaryocytes migrate from their osteoblastic niche towards the vascular niche, where pro-platelets are formed and shed into the blood stream [22]. This theory has gained support from direct in vivo visualization by video-microscopy in the bone marrow of the pro-platelet formation and platelet shedding by megakaryocytes, located in close contact with vascular endothelium [23]. Early on, it was determined that fibrillar collagen supports MK maturation and proplatelet formation in vitro [24]. α2β1 has been found to be critcally required for stress fiber formation in the maturing MK, which is considered an important process for proplatelet formation [25]. Recent experiments using retroviral vectors in a mouse model demonstrated a ligand-dependent down regulation of the activated α2 integrin during megakaryocyte maturation in a collagen rich environment [26]. Also, in WASp (Wiscott-Aldrich Syndrome protein)-deficient mice, a defect was demonstrated in the negative regulation of proplatelet formation mediated by the α2 integrin [27]. Furthermore, *ANKRD26* mutations in patients with type 2 familial thrombocytopenia were associated with both reduced α2 integrin expression and decreased mean platelet volume [28].

Systemic murine *Itga2* knockouts did not result in thrombocytopenia [9], [10], which was interpreted to mean that α2β1 is not involved in MK and proplatelet formation, in contrast to the wealth of data from other laboratories showing that in the bone marrow niche, the binding of MK α2β1 to type I collagen and subsequent activation of the Rho-ROCK pathway delays proplatelet formation [6], [7], [8]. While our results in the conditional MK *Itga2* knockout mouse confirm that α2β1 is probably not essential for MK development and platelet production, they are also consistent with the general conclusion that inhibition of MK α2β1 adhesion to collagen in the bone affects the timing of proplatelet formation reflected in decreased MPV. Unfortunately, neither of the previous studies of systemic knockout mice measured MPV.

The creation of the floxed-*Itga2* mouse will now enable investigators to distinguish the tissue-specific effects of variation in α2β1 alone or in combination with other receptors by a variety of cells, including platelets, peripheral blood mononuclear cells, endothelial cells or smooth muscle (mural) cells. Given the ubiquitous expression of α2β1, there are many opportunities in which the study of conditional knockout mice will generate meaningful in vivo data. For example, α2β1 is expressed by activated neutrophils and mediates their translocation to the extravascular space. Constitutive knockout of neutrophil versus fibroblast or smooth muscle cell expression of α2β1 would generate a model to compare the relative contribution of each cell type to the processes of acute inflammation and wound healing [29]. Similar approaches could be employed to distinguish the contribution of α2β1 in vivo to tubular morphogenesis of epithelial structures [9], development of the renal tubular system [30], and tumorigenesis or metastasis of numerous cancer cell types [31], [32].

In summary, we show that mice lacking MK α2β1 develop normally, are fertile, and like their systemic α2β1 KO counterparts, exhibit no significant defects in platelet function. These results corroborate prior findings in systemic α2β1 knockout mice and confirm that the absence of α2β1 in MK of those mice was largely if not exclusively responsible for the observed platelet phenotype. The only significant difference in our hands was a reduction in mean platelet volume, which is consistent with the reported involvement of α2β1 in MK maturation and rate of proplatelet formation in the bone marrow.
